# The Influence of Selected Titanium Alloy Micro-Texture Parameters on Bacterial Adhesion

**DOI:** 10.3390/ma17194765

**Published:** 2024-09-28

**Authors:** Jolanta Szymańska, Monika Krzywicka, Zbigniew Kobus, Anna Malm, Agnieszka Grzegorczyk

**Affiliations:** 1 Chair of Comprehensive Dentistry, Medical University of Lublin, 20-059 Lublin, Poland; jolanta.szymanska@umlub.pl; 2 Department of Technology Fundamentals, University of Life Sciences in Lublin, 20-950 Lublin, Poland; zbigniew.kobus@up.lublin.pl; 3 Chair and Department of Pharmaceutical Microbiology with Laboratory for Microbiological Diagnostics, Medical University of Lublin, 20-059 Lublin, Poland; anna.malm@umlub.pl (A.M.); agnieszka.grzegorczyk@umlub.pl (A.G.)

**Keywords:** laser surface texturing, titanium alloys, microorganism colonization, biofilm

## Abstract

The colonization of microbes and the resulting formation of biofilms on dental implants are significant contributors to peri-implantitis and the failure of these implants. The aim of the research was to analyze the impact of density and depth of laser texturing of the Ti-6Al-7Nb alloy surface on the colonization of selected microorganisms and biofilm formation. Standard strains of Gram-negative and Gram-positive bacteria and yeasts from the American Type Culture Collection—ATCC—were used to demonstrate the ability to form single-species biofilms in vitro. The study evaluated three types of titanium samples with different texture density and depth. The colonization and biofilm formation abilities of the tested microorganisms were assessed. The obtained results were subjected to statistical analysis. Among the analyzed strains, *L. rhamnosus* showed the highest colonization of the tested surfaces. It was found that there is no relationship between the texture parameters and the number of colony-forming units (CFU/mL) for *C. albicans*, *S. mutans,* and *L. rhamnosus*. For the *F. nucleatum* strain, it was shown that the number of colony-forming bacteria is related to the texture density.

## 1. Introduction

The final stage of prosthetic treatment using implants of missing teeth involves the proper functioning of the stomatognathic system and an aesthetic appearance [1]. Biofilm—a multi-microbial formation that consists mainly of bacteria, as well as protozoa, viruses and fungi—is constantly present in the oral cavity. Biofilm contains up to 100 bacterial species [2]. It is found on hard and soft tissues of the oral cavity, as well as on surfaces like orthodontic bands, clear aligners or dentures [3,4]. The presence of supragingival and subgingival biofilm can be the cause of the evolution of periodontitis and peri-implantitis—polymicrobial inflammatory diseases that cause the destruction of the tissue supporting the tooth/implant; the inflammation of the mucous membrane around the implant (peri-implant mucositis); and/or the inflammation of the tissues involved in the osseointegration of the implant (peri-implantitis) [4].

Both the bone and the surrounding soft tissues (epithelium, gingival connective tissue) are involved in the process of implant osseointegration, and the presence of opportunistic pathogens in the biofilm on the mentioned tissues around the implants and on their surface, including microcracks in the implant surface, disturbs this process. The consequence of the inflammatory cascade caused by microorganisms in the biofilm is the destruction of supporting tissues [5,6,7].

A short adhesion time and intensive multiplication of microorganisms, including opportunistic pathogens, cause the formation of a biofilm, which is a favorable environment for the survival of microorganisms and the maintenance of infections [2]. Modern methods of microbiological identification have allowed for the detection of the presence of some common oral pathogens around the implants, such as *Tannerella forsythia*, *Porphyromonas gingivalis* (*P. gingivalis*), *Prevotella intermedia* (*P. intermedia*) and *Fusobacterium nucleatum* (*F. nucleatum*) [8,9].

The “early colonizers” are mainly considered to be Gram-positive aerobic bacteria—*Streptococcus* spp. and *Actinomyces* spp., which influence the local environment around the implant, making it suitable for “secondary colonizers” such as *F. nucleatum*. This bacterium acts as a “bridge species”. Through coaggregation, it facilitates the adhesion of “late colonizers” and periopathogens, such as *P. gingivalis* [10]. Studies have shown that *P. gingivalis* and *P. intermedia* are mainly accountable for peri-implantitis among “late colonizers” [11]. Severe peri-implantitis may be followed by the loss of the implant at various times. In cases of both early and late implant loss, *F. nucleatum* and *P. gingivalis* were prevalent. Implants that were lost later exhibited greater bacterial diversity and had higher levels of *Treponema*, *Fretibacterium*, *Pseudoramibacter*, and *Desulfobulbus*. In contrast, the microbial communities associated with implants that experienced early loss were highly variable and did not display any significantly more abundant bacterial taxa [12].

The physicochemical properties of the surface of the implants like surface roughness, hydrophobicity, surface free energy, and surface electrochemistry, may influence the formation of the bacterial biofilm [13]. The surface roughness, a common feature of the implant, is one of the factors contributing to the greater colonization and formation of a multimicrobial biofilm [14].

The surface modification of titanium biomaterials plays an important role in the success of surgical procedures, including dental implants. The use of surface engineering methods allows us to obtain the desired functional properties and biological functions. In the past few years, there has been increased interest in laser processing, such as laser ablation, laser-induced periodic surface structures—LIPSS, laser melting, direct laser interference patterning—DLIP, and matrix-assisted pulsed evaporation—MAPLE [15]. Laser technology is also used for surface texturing. Laser surface texturing has numerous advantages, such as high efficiency, ease of operation, environmental friendliness, and the ability to produce controlled and repeatable geometries [16,17,18]. Laser surface texturing has a significant impact on the moisturizing properties and adhesion of bacteria, which is crucial for biomedical applications [19,20]. An important research problem is the selection of appropriate texture parameters, such as the shape and size of the produced dimples and density [21].

Based on the literature review, it was determined that no detailed analysis of the correlation between texture parameters, such as the density and depth of individual texture elements and the adhesion of *Candida albicans* (*C. albicans*), *Streptococcus mutans* (*S. mutans*), *Lactobacillus rhamnosus* (*L. rhamnosus*), *Fusobacterium nucleatum* (*F. nucleatum*) has been performed. Therefore, the novelty of our research is the analysis of the relationship between texture parameters and the adhesion of selected bacteria. The aim of this article is to analyze the effect of selected parameters (density, depth) of the texture of the surface of the Ti-6Al-7Nb alloy on the number of adhering microorganisms.

## 2. Materials and Methods

### 2.1. Laser Surface Texture Preparation

The samples made of Ti-6Al-7Nb titanium alloy (ChM sp. z o.o., Juchnowiec Kościelny, Poland) were the subject of the research. The chemical composition of the alloy complied with ISO 5832-11. Table 1 shows the chemical composition of the alloy.

The surface roughness (Ra) of the samples was 0.06 µm. The laser surface texturing process was carried out as described in the work by Krzywicka et al. [19]. Laser surface texturing was performed in argon shield at 100% power, and the surface scanning was carried out with a laser beam, with a speed of 50 mm/s. After scanning the surface twice with a laser beam with the frequency of 80 kHz, individual textured elements with the depth of approx. 5 μm were obtained. Depths of about 78 μm were obtained using a frequency of 100 kHz, and the surface was scanned 35 times.

In order to determine the number of adhering microorganisms (CFU/mL), dimples with the following density and depth were produced:

–50%, 5 μm,

–10%, 5 μm,

–10%, 78 μm.

The diameter of the dimples was approximately 200 µm. The microgeometric characteristics of the texture were examined utilizing the HIROX KH-8700 digital microscope (Hirox, Tokyo, Japan). Following the laser texturing process, the samples were immersed in distilled water at a temperature of 55 °C within an ultrasonic cleaner (Ultron, Dywity, Poland) for 10 min.

### 2.2. Fungal and Bacterial Cell Culture and Assessment of Different Species of Oral Microbiota Colonization and Biofilm Formation

The following American Type Culture Collection (ATTC) strains were used in the study: *C. albicans* ATCC 10231—yeast-like fungi, *S. mutans* ATCC 25175—Gram-positive bacteria, *L. rhamnosus* GG ATCC 53103—Gram-positive bacteria, and *F. nucleatum* ATCC 25586—Gram-negative bacteria, which may affect biofilm formation on the surface of dental plaque. The microorganisms were used to show the capacity to form in vitro single-species biofilms composed of all the reference strains.

The study was conducted using sterile 6-well polystyrene titration plates (NUNC) and the following reagents: crystal violet, 1% solution (BioMerieux, Warsaw, Poland), ethyl alcohol 96% pure p. a., sterile phosphate-buffered saline (PBS) with calcium and magnesium ions, pH 7.4 ± 0.2, osmolarity 270–290 mOsmol/L (BioMaxima, Lublin, Poland).

#### Preparation of Microorganisms for Analysis

An assessment of the number of adhering microorganisms was carried out (CFU/mL). The prepared bacterial and fungal inocula were transferred into the wells on the 6-well plates containing the appropriate media; the starting densities obtained were 1.5 × 10^7^ CFU/mL (for bacteria) and 5 × 10^5^ CFU/mL fungi. At the same time, the titanium alloy samples were coated with mucin—1% solution (Sigma, Lublin, Poland)—and subsequently placed on the media with a given microorganism suspension, each in a separate well.

The samples were incubated at 35 °C for an hour (adhesion period) under aerobic or anaerobic conditions, depending on the microorganism. After that time, the samples were thoroughly rinsed with 5 mL PBS, placed in fresh sterile media, and incubated at 35 °C under atmospheric conditions suited to each of the microorganisms for 24 h (biofilm formation period) and another 24 h (biofilm maturation). After each 24 h period, the samples were rinsed with 5 mL sterile PBS and the biomaterials were placed in fresh media.

After 48 h incubation, the samples were rinsed once more, placed in test tubes with 3 mL PBS, and shaken at 1000 rpm for 30 min. Next, 10^−1^ to 10^−10^ dilutions were made, plated in the appropriate media, and incubated at 35 °C for 48 h. After incubation, the colonies were counted (Counter Colony Scan 1200; Interscience, Fisher Scientific, Porto Salvo, Portugal) and converted into CFU/mL. At the same time, substrate control and microbial viability control were carried out.

### 2.3. Statistical Analysis

Using Dell Statistica v. 13.1 (Dell Inc., Cracow, Poland, 2016), an examination was carried out to analyze the relationships between variables. Initially, the presence of a correlation between independent variables (density, depth) and dependent variables (CFU/mL) was investigated. Once a correlation was confirmed, a regression function was derived to quantify the relationships. The next step involved verifying the model by checking the assumptions such as the significance of linear regression, the significance of partial regression coefficients, the absence of collinearity between independent variables, homoscedasticity, the absence of autocorrelation of residuals, the normal distribution of residuals, and the expected value of the random component being 0. The data were considered statistically significant at *p* < 0.1.

## 3. Results and Discussion

### 3.1. The Microbial Reference Strains Representing Oral Microbiota Colonization and Biofilm Formation

**Figure 1:**
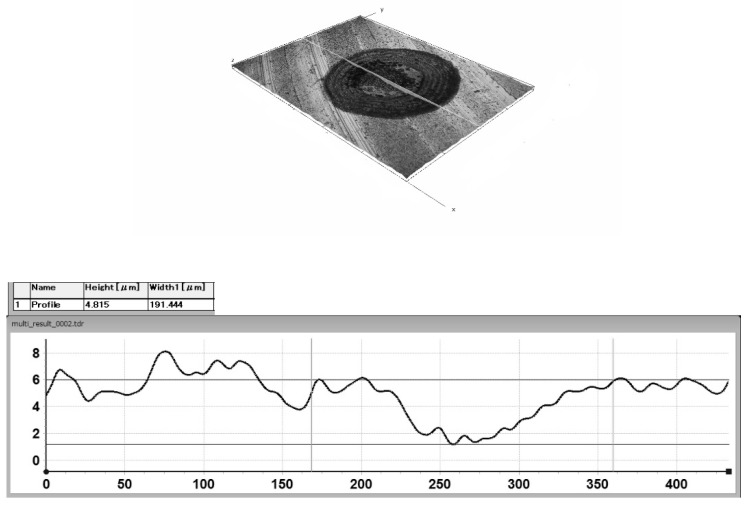
The profile of a single texture element in the form of a dimple with a diameter of 191.44 μm and a depth of 4.82 μm.

**Figure 2:**
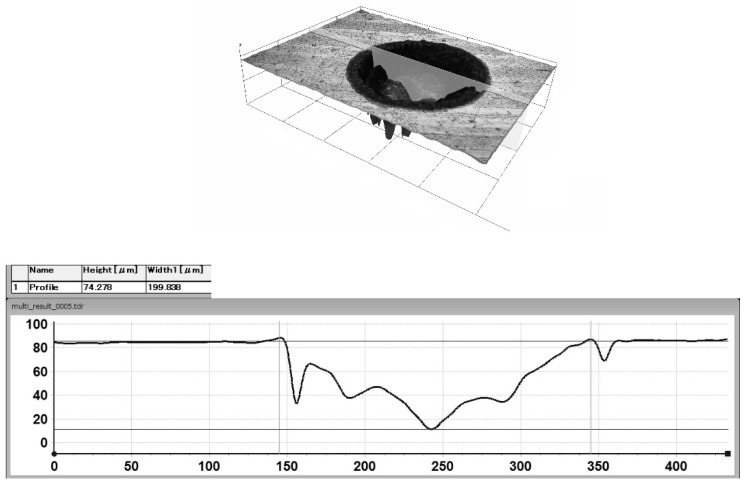
The profile of a single texture element in the form of a dimple with a diameter of 199.84 μm and a depth of 74.28 μm.

The reference sample was not subjected to the laser surface texturing (Ra = 0.06 μm). Figure 1 and Figure 2 show the profiles of the individual texture elements.

Table 2 shows the number of CFU/mL of the microbial references strains representing oral microbiota forming a biofilm on the titanium alloy samples.

All analyzed strains showed the lowest adhesion to the non-textured surface. The lowest adhesion to the Ti-6Al-7Nb surface among all analyzed strains was demonstrated by *S. mutans*, and the highest was demonstrated by *L. rhamnosus*.

*C. albicans* ATCC 10231 showed the lowest biofilm formation on the textured surface, on which dimples of the lowest depth were created. The CFU/mL value for the sample on the surface of which textures with a depth of 5 µm and density of 10% were created is 6.67% higher than the untextured sample. Comparing the biofilm formation on the surface on which dimples of the same depth and different densities were created, it can be seen that a significantly lower CFU/mL value was recorded for the lower density.

*S. mutans* ATCC 25175 showed the lowest biofilm formation on the textured surface on which dimples of the lowest depth were created. The CFU/mL value for the sample on the surface of which textures with a depth of 5 µm and density of 10% were created is 309.8% higher than the untextured sample. Compared to the surface on which dimples of the same depth and different densities were created, it can be seen that a significantly lower CFU/mL value was recorded for the lower density.

*L. rhamnosus* GG ATCC 53103 showed the lowest adhesion to the textured surface on which dimples with a depth of 5 µm and a density of 50% were created. The CFU/mL value for the sample on the surface of which textures with a depth of 5 µm and density of 50% were created is 30% higher compared to the untextured sample. Comparing the colonization and biofilm formation on the surface on which dimples of the same density were created, it can be seen that a lower CFU/mL value was recorded for the higher depth of the dimples.

*F. nucleatum* ATCC 25586 showed the lowest biofilm formation on the textured surface on which dimples with a depth of 5 µm and a density of 10% were created. The CFU/mL value for the sample on the surface of which textures with a depth of 5 µm and density of 10% were created is 77.8% higher than the untextured sample. Comparing the biofilm formation on the surface on which dimples of the same density were created, it can be seen that a lower CFU/mL value was recorded for the lower depth of the dimples.

### 3.2. The Dependence of the Number of Adhering Microorganisms on Texture Geometry

It was found that there is no correlation between the texture parameters and CFU/mL for *C. albicans* ATCC 10231, *S. mutans* ATCC 25175 and *L. rhamnosus* GG ATCC 53103, and therefore no regression function can be found.

For the *F. nucleatum* ATCC 25586 strain, it was shown that the independent variable density is correlated (*p* < 0.1) with the dependent variable CFU/mL. There is a correlation between the studied characteristics. The standard error of the estimate for the intercept about its value is relatively large. The coefficient of determination is 0.9; hence, the model explains 99.9% of the variability in CFU/mL. The linear correlation coefficient R is equal to 0.999; thus, there is an almost completely linear relationship between the dependent variable and the independent variables. Linearity is checked with the F-test. The *p*-value for this test is 0.020, which means that the regression equation is significant. The standard error of the estimation is 56,569, which means that the predicted values of the CFU/mL variable differ from the empirical values by an average of 56,569. The residual distribution is assumed to be normal. The value of the d statistic is 2.5, which implies that there is autocorrelation of the residuals. This may be due to a small number of data (3) or other factors affecting the CFU/mL value. The random component εi has an expected value of 0. The average value of the residuals is equal to 0. The analyses carried out show that sample no. 3, on the surface of which dimples with a depth of 5 µm and a density of 50% were created, has a significantly large impact on the load of the regression equation. This could be the cause of the high standard error value.

The regression equation is:

CFU/mL = −90,000 + 6,100,000 × Density ± 51,962(1)

This means that if the density increases by 1%, the CFU/mL value increases by 6.1 × 10^6^.

The model verification showed that the assumptions of unbiased residuals, random deviations and the lack of autocorrelation of residuals are not met. This could be attributed to a limited data set (3) or other factors affecting the CFU/mL value.

## 4. Discussion

The CFU/mL values were significantly different for individual bacterial strains. The lowest adhesion to the control sample was shown by *S. mutans*. The studies showed increased bacterial adhesion to the textured surface. The highest CFU/mL value for *C. albicans* and *S. mutans* was recorded for the sample on the surface of which textures with a depth of 78 µm and density of 10% were produced, which is 166.7% and 614.8% higher in comparison to the non-textured sample, respectively. The highest CFU/mL value for *L. rhamnosus* was recorded on the surface where the dimples with the lowest density and depth were created, and this value was 109.9% higher than for the control sample. The highest CFU/mL value for *F. nucleatum* was recorded on the surface where the dimples with the lowest depth and highest density were created, and this value was 996.3% higher than for the control sample. The lowest percentage increase in adhesion compared to the control sample was shown by *L. rhamnosus*, which also showed the highest adhesion to the Ti-6Al-7Nb alloy. The lowest CFU/mL value for *C. albicans*, *S. mutans, F. nucleatum* was shown for the lowest depth of a single textural element and the lowest density. The lowest CFU/mL value was recorded for the highest density for *L. rhamnosus* alone. The mechanism of bacterial adhesion is complex. Undoubtedly, laser micromachining causes increased roughness, and thus increases the contact surface. The diameters of all analyzed bacteria were smaller than the distance between the dimples as well as the diameter, and the depth of the generated textures, so it can be assumed that the generated textures promoted bacterial migration, penetration into dimples, and biofilm formation. The most similar results were obtained for *S. mutans* and *C. albicans*, despite these being pathogens from different groups—a Gram-positive bacterium and a fungus, respectively. The sizes of these pathogens also differed significantly, with *C. albicans* measuring 2–4 µm in diameter and *S. mutans* approximately 0.5–0.75 µm in diameter [22]. The most similar sizes were found in *L. rhamnosus* (from 0.8 to 1.0 μm in width) [23] and *S. mutans*, and both strains belong to Gram-positive bacteria. Despite these similarities, extremely different CFU/mL values were obtained.

The available literature reports present divergent results on the effects of laser surface texturing on microorganism adhesion. Similar results to ours were obtained by Singh et al. [24], Esfahanizadeh et al. [25], and Uhlmann et al. [26]. Singh et al. [24] produced fish-scale, octagonal, and hexagonal features with varying densities of 43.4%, 55%, 74.7%, heights of 97 μm, 102 μm, 127 μm, and a diameter/edge of pillar of 135 × 95 μm, 53 μm, 1.06 μm on the surface of the Ti-6Al-4V alloy. An average bacterial area coverage of *Staphylococcus aureus* (*S. aureus*) could be approximated to 35% for untextured and to 60% for textured. Singh et al. [24] also indicate that the size of the textures has an influence on bacterial adhesion. Esfahanizadeh et al. [25] report that a higher pathogen count was found on the surface of the treated titanium (with similar microgrooves at 8 μm intervals) compared to the untreated sample. The mean count of *Aggregatibacter actinomycetemcomitans* was 11.3163 and 9.6941 log CFUs/mL, with 11.3437 and 10.0831 log CFUs/mL for *P. intermedia*, and 12.1176 and 10.1213 log CFUs/mL for *P. gingivalis* in the laser and titanium groups, respectively. Uhlmann et al. [26] produced textures of various parameters on the surface of the Ti-6Al-4V alloy and showed that the adhesion of *S. mutans* to the surface on which the LIPSS was made, with a height of 300 nm, a microcavity width of 15 μm, a length of 30 μm, a height of 5 μm, and a microcavity length of 15 μm (rest unchanged), showed similar results for bacterial attachment, and these values are significantly higher than for the control sample subjected to chemical polishing. Based on the conducted research and the results of the above-mentioned authors [24,25,26], the production of textures with the size of single micrometers will promote the adhesion of various bacterial strains.

Grössner-Schreiber et al. [27] performed a Ti Grade 2 laser treatment and found no difference in the adhesion of *S. mutans* and *Streptococcus sanguis* between polished Ti and Ti laser. Hauser-Gerspach et al. [28] treated the surface of commercially pure titanium using different laser energy densities (12.74 J/cm^2^ and 63.69 J/cm^2^). They showed no differences in the adhesion of *Streptococcus sanguinis* and *P. gingivalis* bacteria between laser-processed and polished samples. Du et al. [29] produced the LIPSS on the Ti Grade 4 surface, which showed no statistically significant difference in OD (optical density) between the polished surface (*Escherichia coli* (*E. coli*)*:* 0.973; *S. aureus*: 0.971) and the LIPSS surface (*E. coli:* 0.969; *S. aureus*: 0.955). It is worth emphasizing that Grössner-Schreiber et al. [27], Hauser-Gerspach et al. [28], and Du et al. [29] do not provide the sizes of the textures produced, so it is impossible to compare them with the results of our research and the size of the analyzed bacteria. Many authors obtained different results and showed that laser surface texturing reduces bacterial adhesion [30,31,32,33,34,35,36]. Parmar et al. [30] produced micro-pits of various diameters (24–35 μm) and depths (4.58–78 μm) on the surface of the Ti-6Al-4V alloy. In our studies, a depth of 78 µm was also analyzed, but with a much larger diameter of 200 and other pathogens and alloys. The studies carried out showed a 75% decrease in the adhesion of *S. aureus* compared to the untreated sample. Additionally, in the case of the textured sample, a reduction in CFU/mL by 80% was demonstrated after 72 h, compared to a 20% reduction for the non-textured sample. Shiju et al. [31] performed laser texturing of the Ti-6Al-4V alloy surface and produced dimples with a depth of 2.5 μm and a diameter of 10 μm. The textures produced are significantly smaller in size than those produced in our studies, but are still larger than the size of the bacteria when using confocal microscopy; it was shown that *S. aureus* adhesion was lower on the surface of treated samples than on untreated samples [31]. Doll et al. [32] carried out the laser processing of titanium, on the surface of which three different types of Sharklet™-like textures were produced (grooves with a width of 2 μm, a depth of ≥2 μm, and various lengths from 4 μm to 16 μm, arranged in a periodic diamond-like pattern at fixed intervals 2 µm between elements), with linear grooves (2 µm wide, ≥2 µm deep and a fixed interval of 2 µm) and grid structures (an orthogonal overlap of two structural grooves with the same dimensions as above). The analysis showed the reduced adhesion of *S. aureus* on all tested microstructures compared to smooth titanium surfaces. Based on the studies conducted by Doll et al. [32], who also produced micrometer-sized textures, larger than the diameter of *S. aureus*, it can be concluded that not only the size but also the shape of the produced textures plays an important role in limiting bacterial adhesion. Eghbali et al. [33] proved that the surface modification of the Ti-6Al-4V alloy at depths ranging from 0.5 to 50 μm inhibits *E. coli* adhesion when using a higher laser frequency of 160 kHz. However, increasing the groove distances to over 50 μm and using a lower laser frequency of 20 kHz decreases laser pulse overlaps, leading to enhanced cell adhesion. Chik et al. [34] produced LIPSS (pulse duration 380 fs, power 0.11 W, wavelength 515 μm, frequency 200 kHz) on the surface of Ti Grade 5. Laser-treated surfaces were characterized by lower bacterial adhesion compared to polished surfaces (by >80% in the case of *E. coli* and >20% in the case of *S. aureus*). Our experiments, as well as the studies by Chik et al. [34], showed that individual bacterial strains adhere to a given surface to a varying extent. Zwahr et al. [35] produced crater-like structures on the Ti Grade 4 surface (separation distance of 50 μm), then, directly on this texture, a hole-like pattern with a 5 μm spatial period was generated. The adhesion of *E. coli* bacteria was reduced by 30% compared to the control sample. Research conducted by Zwahr et al. [35] indicated that the appropriately selected shapes of textures, even micrometer-sized, can limit the adhesion of bacteria. Yao et al. [36] subjected Ti to laser processing and produced a circular pattern on the surface of the samples. The laser-treated samples exhibited lower roughness than the control disks subjected to autoclaving treatments and were characterized by slightly less *P. gingivalis* adhesion and less *P. gingivalis* colonization than the control samples. Yao et al. [36] did not provide texture sizes; hence, it is impossible to compare them with our research results.

The authors [37,38,39,40] who produced textures with sizes below 1 micrometer, including nanotextures, showed reduced bacterial adhesion. Orazi et al. [37], on the surface of the Ti-6Al-4V alloy, using different laser operating parameters (the first treatment—the pulse energy of 16 µJ and an overall dose of 27 J/cm^2^, the second treatment—the pulse energy of 32 µJ and an overall dose of 270 J/cm^2^), created an LIPSS with dimensions of 100–200 nm. Based on the conducted research, it was shown that laser-treated surfaces exhibit lower *S. aureus* adhesion (2 log_10_ CFU) compared to the untreated surface (5 log_10_ CFU). Meinshausen et al. [38] created wavy textures on the surface of Ti Grade 4 with a spacing of 0.7 to 5 µm and a height of 100 to 800 nm, and demonstrated that laser-treated surfaces show a lower adhesion of *S. aureus* bacteria compared to the control sample. Luo et al. [39] conducted laser treatment on 99.7% pure titanium and created three types of nano-ripples on the surface of the samples: LIPSS (400 nm), columns with overlapped LIPSS, and similar periods with LIPSS, but with nano-ripples interrupted by shallow sinuous grooves (1 μm pitch) vertically. It was shown that the tested textures could prevent bacterial colonization and biofilm formation, and their antibacterial effectiveness against *E. coli* ranged from 43% to 56%. Donaghy et al. [40] produced a spiky surface and decreasing peak-to-peak distance between ripples (0.63 to 0.315 µm) on the Ti-35Nb-7Zr-6Ta surface, and showed a significant reduction in *S. aureus* adhesion on the treated surface compared to the control sample.

The presented research results indicate that the laser texturing of the surface of titanium alloys requires further optimization. In particular, it is necessary to take into account the species of bacteria that form biofilms and which, as proved by the research results, show varying adhesion to the surfaces of alloys used for implants.

## 5. Conclusions

Laser surface texturing has an impact on microorganism colonization and biofilm formation. Drawing from the research carried out, it can be concluded that different species of oral microbiota react in varying degrees to the produced textures. *C. albicans*, *S. mutans*, *F. nucleatum* showed the lowest colonization and biofilm formation to the lowest depth textures, while *L. rhamnosus* demonstrated the lowest depth but with different densities—50% for *L. rhamnosus*. Among the analyzed strains, *L. rhamnosus* showed the highest adhesion to the tested surfaces. It was found that there is no relationship between texture parameters and the CFU/mL value for *C. albicans, S. mutans* and *L. rhamnosus*. For the *F. nucleatum* strain, it was shown that the number of colony-forming bacteria is related to the texture density.

There is a need to continue research on micro- and nanotextures of titanium surfaces for dental implants, which minimize colonization by microorganisms and thus limit the development of inflammatory processes around implants, promoting the proper course of osteointegration.

## Figures and Tables

**Table 1:** The chemical composition of Ti-6Al-7Nb.

Fe	O	N	C	H	Al	Nb	Ta	Ti
max. 0.25%	max. 0.2%	max. 0.05%	max. 0.08%	max. 0.009%	5.5–6.5%	6.5–7.5%	max. 0.5%	rest

**Table 2:** The number of CFU/mL of the microbial references strains representing oral microbiota forming a biofilm on the titanium alloy samples.

Microbial Species	Sample	Biofilm Size	
		CFU/mL	log CFU/mL
*C. albicans* ATCC 10231	Model	0.75 × 10^4^ ± 0.13 × 10^4^	3.88 ± 3.11
	10%, 5 μm	0.8 × 10^4^ ± 0.15 × 10^4^	3.90 ± 3.17
	10%, 78 μm	2.0 × 10^4^ ± 0.36 × 10^4^	4.30 ± 3.55
	50%, 5 μm	1.7 × 10^4^ ± 0.53 × 10^4^	4.23 ± 3.72
*S. mutans* ATCC 25175	Model	0.61 × 10^4^ ± 0.23 × 10^4^	3.78 ± 3.36
	10%, 5 μm	2.50 × 10^4^ ± 0.36 × 10^4^	4.39 ± 3.55
	10%, 78 μm	4.36 × 10^4^ ± 1.7 × 10^4^	4.63 ± 4.23
	50%, 5 μm	3.05 × 10^4^ ± 1.9 × 10^4^	4.48 ± 4.28
*L. rhamnosus* ATCC 53103	Model	2.13 × 10^7^ ± 1.09 × 10^7^	7.32 ± 7.03
	10%, 5 μm	4.47 × 10^7^ ± 2.4 × 10^7^	7.65 ± 7.38
	10%, 78 μm	3.36 × 10^7^ ± 1.25 × 10^7^	7.53 ± 7.10
	50%, 5 μm	2.77 × 10^7^ ± 1.5 × 10^7^	7.44 ± 7.17
*F. nucleatum* ATCC 25586	Model	0.27 × 10^6^ ± 0.10 × 10^6^	5.43± 5.00
	10%, 5 μm	0.48 × 10^6^ ± 0.19 × 10^6^	5.68 ± 5.28
	10%, 78 μm	0.56 × 10^6^ ± 0.19 ×10^6^	5.74 ± 5.28
	50%, 5 μm	2.96 × 10^6^ ± 0.94 × 10^6^	6.47 ± 5.97

## Data Availability

The original contributions presented in the study are included in the article, further inquiries can be directed to the corresponding author.
